# Impacts of the COVID-19 Pandemic and Self-Isolation on Students and Staff in Higher Education: A Qualitative Study

**DOI:** 10.3390/ijerph182010675

**Published:** 2021-10-12

**Authors:** Holly Knight, Sophie Carlisle, Mórna O’Connor, Lydia Briggs, Lauren Fothergill, Amani Al-Oraibi, Mehmet Yildirim, Joanne R. Morling, Jessica Corner, Jonathan Ball, Chris Denning, Kavita Vedhara, Holly Blake

**Affiliations:** 1 School of Medicine, University of Nottingham, Nottingham NG7 2UH, UK; sophie.carlisle@nottingham.ac.uk (S.C.); joanne.morling@nottingham.ac.uk (J.R.M.); chris.denning@nottingham.ac.uk (C.D.); kavita.vedhara@nottingham.ac.uk (K.V.); 2 School of Health Sciences, University of Nottingham, Nottingham NG7 2HA, UK; morna.oconnor@nottingham.ac.uk (M.O.); lydia.briggs@nottingham.ac.uk (L.B.); lauren.fothergill@nottingham.ac.uk (L.F.); amani.al-oraibi@nottingham.ac.uk (A.A.-O.); mehmet.yildirim@nottingham.ac.uk (M.Y.); holly.blake@nottingham.ac.uk (H.B.); 3 NIHR Nottingham Biomedical Research Centre, Nottingham University Hospitals NHS Trust and the University of Nottingham, Nottingham NG7 2UH, UK; 4 University Executive Board, University of Nottingham, Nottingham NG7 2RD, UK; jessica.corner@nottingham.ac.uk; 5 Biodiscovery Institute, University of Nottingham, Nottingham NG7 2RD, UK; jonathan.ball@nottingham.ac.uk; 6 School of Life Sciences, University of Nottingham, Nottingham NG7 2UH, UK

**Keywords:** COVID-19, SARS-CoV-2, coronavirus, workplace, workforce, social isolation, mental health, students, staff, focus groups, semi-structured interviews, qualitative

## Abstract

This qualitative study explored the impact of COVID-19 self-isolation and social restriction measures on university students, through the perspectives of both students and the staff supporting them. The study comprised 11 focus groups (students) and 26 individual interviews (staff) at a higher education institution in England during a period of national lockdown (January–March 2021). Participants were university students (*n* = 52) with self-isolation experiences and university staff (*n* = 26) with student-facing support roles. Focus group and interview data were combined and analysed using an inductive thematic approach. Four themes emerged: ‘Adaptation during the pandemic’, ‘Practical, environmental, and emotional challenges of self-isolating’, ‘Social factors and their impact on COVID-19 testing and self-isolation adherence’, and ‘Supporting self-isolation’. Students and staff struggled with the imposed restrictions and shift to online education. Students found it difficult to adapt to new expectations for university life and reported missing out on professional and social experiences. Students and staff noted concerns about the impact of online teaching on educational outcomes. Students endorsed varied emotional responses to self-isolation; some felt unaffected whilst others experienced lowered mood and loneliness. Students were motivated by pro-social attitudes; campaigns targeting these factors may encourage continued engagement in protective behaviours. Staff struggled to manage their increased workloads delivering support for self-isolating students. Universities must consider the support needs of students during self-isolation and prepare for the long-term impacts of the pandemic on student wellbeing and educational attainment. Greater support should be provided for staff during transitional periods, with ongoing monitoring of workforce stress levels warranted.

## 1. Introduction

The ongoing SARS-CoV-2 (COVID-19) pandemic has triggered national and global restrictive measures in an effort to contain the virus. In the UK, measures such as stay-at-home orders, social distancing, and travel restrictions have been implemented at multiple points over the course of the pandemic, impacting the higher education sector substantially [1]. Despite returning home early in the pandemic, subsequent reopening of university campuses for the new academic year (September/October 2020) led to widescale student movement across the country and coincided with a second surge of COVID-19 within the UK [2]. The introduction of a national tiered system in October 2020 brought rapidly changing social restrictions, impacting not only the delivery of education, but also the mechanics of the wider university workforce. As well as abrupt changes to their method of learning, students have faced changes to their exams, placements, and electives [3]. Both home and international students have contended with prolonged separation from family and friends, with many being required to self-isolate in the confinement of their room or home. In parallel, academic staff have also been faced with sudden and ongoing changes to the delivery of teaching, requiring the adoption of remote working, ensuring in-person teaching is delivered in a COVID-safe way, and the frequent adaptation of materials, all with very often minimal notice and preparation time. This has resulted in an increase in workload related to the ever-changing guidance, restrictions, and support needs of students [4].

In addition to the rapid transition to online teaching, multiple universities developed capacity to conduct mass asymptomatic COVID-19 testing programmes in an effort to combat transmission between students [5,6]. Successful mass testing relies not only on testing uptake, but also on adherence to containment strategies, such as self-isolation, following a positive result [7,8]. ONS data suggest high levels of adherence to social distancing and isolation behaviours in university students [9], with Nixon and colleagues reporting that 99% of their student sample testing positive in the prior fortnight had been self-isolating [10]. It has been suggested that adherence to self-isolation may be more heavily impacted by the availability of resources rather than psychological motivation [11]. Other factors, including a drive to socialise and personal concern about the mental health implications of isolation, may also contribute to students’ willingness to isolate [12]. Whilst there has been a deluge of research studying the effects of the COVID-19 pandemic on mental health in the general population, there is less specifically focusing on university students and staff. Further, of the research that does exist, the evidence points to wide-ranging negative impacts on wellbeing, including significantly lower mood [13], reduced mental wellbeing and increased stress [14], and increased depression [15]. There is even less information on the impact of self-isolation on students and the staff supporting them. At present, mental health problems are a primary cause of sickness absence in the UK, accounting for 17.5 million days of lost workforce productivity each year [16]. University students represent a significant proportion of the future workforce and, as a result, understanding the impact of the COVID-19 pandemic and self-isolation measures on students’ wellbeing is paramount.

This qualitative study was conducted at a large, campus university in central England (University of Nottingham), which implemented mass asymptomatic testing and recorded a large increase in the number of positive COVID-19 cases and, therefore, self-isolation during the Autumn term (2020). The aim of this study was to explore the impact of the COVID-19 pandemic on university students from the perspectives of both students and the staff supporting them, with particular emphasis on the wellbeing and support needs of students who were required to self-isolate. Our intention is to reflect upon the experiences of students and staff, informing future policy and practice in the context of university settings as we continue to navigate the COVID-19 pandemic.

## 2. Materials and Methods

### 2.1. Study Design

This qualitative study included focus groups with students and individual interviews with university staff members from the University of Nottingham campuses in central England. This institution has the ninth largest student body of all British academic institutions [17] with both on and off-campus accommodation and communal living spaces to those in on-campus housing. The institute provides both undergraduate and graduate taught and research courses. A total of 11 online focus groups with 52 student participants and 26 individual interviews with staff members were conducted. The study design aligned with the consolidated criteria for reporting qualitative studies (COREQ) guidelines [18] (Supplementary File S2). The study protocol was approved by the Research Ethics Committee of the University of Nottingham Faculty of Medicine and Health Sciences (Ref: FMHS 76-0920).

### 2.2. Study Context

This study took place between January and March 2021 at the beginning of Spring term, starting shortly after the third national lockdown was announced. During this time, national restrictions were in place, limiting movement outside of the home unless for work that could not be done at home, exercise once per day, and to get essential items such as food. Additionally, household mixing was restricted, and there were restrictions on travel, access to facilities and services. Easing of national restrictions did not begin until after data collection was completed.

### 2.3. Participants, Recruitment and Sampling

Focus group participants were university students who had experienced a period of self-isolation for any reason, including receiving a positive test, being in contact with someone who has tested positive, or pre-cautionary self-isolation. Recruitment occurred through two routes: (1) Students who had taken part in an established cohort study [19], and (2) Students who had received a positive COVID-19 test result through the university medical centre and had, therefore, undergone a period of self-isolation. Both home and international students were recruited. Recruitment took place between January and February 2021. Students received a £20 voucher as compensation for their participation.

University staff were purposively sampled for one-to-one interviews, to ensure a variety of roles within the university were represented. Specifically, staff with student-facing roles, or a role relating to COVID-19 strategies or student support were invited to interview, including staff in teaching and/or research, leadership, support, and pastoral care roles. Individual interviews were offered to ensure staff were able to attend at a time that was convenient to them, bolstering uptake. Recruitment took place between January and March 2021. Students and staff provided both written and verbal informed consent. Sample characteristics are provided in Table 1 and Table 2.

### 2.4. Qualitative Interviews and Focus Groups

Given the rapidity of the required data collection, we hosted a total of 11 focus groups, including a mix of smaller groups of between two to four participants, larger groups of between five and eight participants and one individual interview with a single participant (due to non-attendance). Focus groups were held online using video-conferencing facilities (Microsoft Teams). Online data collection was necessary due to the national lockdown imposed at the time, as well as social isolation policy. This form of data collection has been commonly used as means of improving access for geographically dispersed or hard-to-reach populations [20]. All focus groups took place within a period of three weeks in January and February 2021. Focus groups lasted for 54 to 81 min (mean 70 min). Two psychologists generated the question guide (HB, HK, Supplementary File S3) and focus groups were facilitated and moderated by two PhD students who were part of the research team (SC, MO). All focus groups were conducted in line with NHS England guidance on focus group delivery and followed the same questioning route [21]. All members of the team were trained in qualitative research and interview skills.

A total of 26 staff interviews were conducted via online video-conferencing facilities (Microsoft Teams), lasting between 21 and 100 min (mean 65 min). Recruitment for the staff interviews was high to ensure representation of the range of student facing roles and recruitment across all role families continued until data saturation occurred. The individual interview modality was chosen for staff data collection to encourage openness and to enhance uptake by allowing flexible scheduling. Interviews took place between January and March 2021. A separate question guide was used that had been generated by the same two psychologists (HB, HK). Interviews were carried out by two PhD students (MO, LF) and followed the same questioning route. All student focus groups and staff interviews were audio and video recorded and transcribed verbatim. To expedite the process, transcription was conducted by both a professional transcription company and by two members of the research team (MO, SC), with all transcripts checked for accuracy.

### 2.5. Data-Analysis

Data were analysed using inductive thematic analysis [22], which benefits from theoretical flexibility and simplicity, and the ability to identify, organise, and describe the experiences of students and staff through the identification of key themes and subthemes. Focus group and individual interview data were reviewed separately in the first instance to identify unique factors and commonalities in the data, with both datasets subsequently combined to create a nuanced understanding of self-isolation experiences. One researcher (LB) conducted a preliminary scan of the student focus group and staff interview data, allowing generation of initial codes for data extracts. To ensure reliability of the data analysis, a second researcher (HK) independently examined the emerging coding, allowing further refinement. Codes and subcodes were subsequently grouped into themes, ensuring that the data cohered together meaningfully, whilst themes were clear and distinct. Finally, a detailed analysis was conducted for each theme and data excerpts were identified to illustrate the final themes. Combining data sources allowed iterative triangulation of an initial coding scheme using individual and group level input. Staff interviews provided additional contextual information, improving interpretation of the focus group data and providing stronger understanding of the academic landscape during the pandemic. Convergence of focus group and individual interview data enhanced the reliability of the coding scheme [23]. Both researchers (LB, HK) reviewed the final thematic outcomes. NVivo 11 (QSR International Ltd., Melbourne, Australia) was used as a data management tool throughout data analysis.

## 3. Results

Four key themes were interpreted from the combined staff and student data: adaptation during the pandemic; the practical, environmental, and emotional challenges of self-isolation; social factors and their impact on COVID-19 testing and self-isolation adherence; and supporting self-isolation. A thematic map illustrating the relationships between the key themes and subthemes is provided in Supplementary File S4. Table 3 shows the list of all the key themes and subthemes and representative quotes. A description of each theme and subtheme, along with representative quotes is presented in more detail in Supplementary File S1.

### 3.1. Adaptation during the Pandemic

#### 3.1.1. A New Normal

Many students reported initial difficulties adapting to periods of self-isolation, particularly because they felt the lines between self-isolation and lockdown became blurred due to national restriction measures. This sentiment was exacerbated for students living in halls, who were often exposed to extended periods of isolation with other students due to high case rates. For many, these lockdown measures elicited changes to their sense of normal.

#### 3.1.2. Challenges to Adaptation

The challenges of adapting to the pandemic were multifactorial for students and staff, but many reported that these challenges reduced as the pandemic progressed and they adjusted to meet the new normal. For students, challenges centred around disruptions to their studies (particularly as a result of self-isolation), which required increased flexibility from the university and variable teaching methods. Some staff felt that the online teaching modality had created a unique cohort of students with unique needs, with multiple students reporting that they felt disadvantaged by the new learning environment.

A broader challenge was the discrepancy between students’ expectations of a normal university experience and the infringements placed on this by national measures, particularly for first-year students. For staff, there was a similar adjustment period to the variable teaching methods and different ways of working, alongside trying to meet the expectations of students (which caused students to drop out in some cases).

For staff in student-facing roles, there was a general concern about contracting COVID-19, despite being provided with protective equipment. Although the transition was mostly well received, multiple staff members noted that ground staff did not feel their own health and wellbeing was sufficiently supported, creating anxiety and the sense that the university was putting their staff “in jeopardy”.

### 3.2. The Practical, Environmental and Emotional Challenges of Self-Isolating

#### 3.2.1. Self-Isolation Environment

The environment in which students self-isolated was a significant challenge for many, particularly for those confined to small rooms with no outdoor spaces. Feelings of boredom and entrapment were common.

The restrictions presented by being required to isolate within a household also had broader impacts, affecting students’ relationships with other housemates (both positively and negatively). Despite this, both students and staff generally felt it was more positive to self-isolate with others rather than alone.

From a staff perspective, most appreciated the role that environment played in coping with self-isolation, particularly for students living in university accommodation. For staff working with international students, there was recognition that self-isolation presented nuanced challenges, particularly for new international students without existing social networks or supports.

#### 3.2.2. Emotional Effects of Self-Isolation

The emotional effects of self-isolation were variable; some students reported feeling unaffected by self-isolation, whilst others reported experiencing low mood, loneliness, frustration, and a lack of purpose. Many students reported notable decreases to their motivation due to the disruption that self-isolation had on their daily routines and social connectedness. Some students also described feeling guilty if they had received a positive COVID-19 test prompting isolation, given that this would impact others in their accommodation.

#### 3.2.3. Self-Isolation Impacts on University Service Provision

Staff reported being significantly impacted by students’ self-isolation. This included dealing with frustrated or non-compliant students, ensuring those who required greater mental health input were cared for, and outreach to students who were self-isolating regularly. Staff noted this added significant burden to their workloads and for some, affected their mood. These experiences were further exacerbated by external pressures, such as adherence to government regulations and balancing this with recognition of the mental health impacts that isolation had on students. In some cases, staff reported “firefighting” throughout the pandemic, being unable to do their usual work, or make developments in other areas of their role due to these additional pressures. It was also noted that additional support for staff would be welcomed, with one staff member feeling that there had been limited support provided.

### 3.3. Social Factors and Their Impact on COVID-19 Testing and Self-Isolation Adherence

#### 3.3.1. Testing: Social Factors, Barriers and Enablers

Many students felt that frequent asymptomatic testing was useful. However, some staff reported groups of students who refused to participate in testing because they feared repercussions (such as having to self-isolate, being blamed for making others isolate, or being unable to go home for Christmas). Students and staff felt that greater flexibility in testing delivery and incentives might improve adherence.

#### 3.3.2. Compliance with Self-Isolation

Almost all students in the study described feeling that self-isolation was important but acknowledged that some students did not comply with regulations. Many factors appeared to affect students’ self-isolation adherence behaviours, and this variability was recognised by staff. These factors included: moral views about adherence (the ‘right’ thing to do), lack of understanding of the rules, protecting others, rules not being enforced by the university, peer pressure, fear of missing out on social activities, inconsistent or confusing communications, and repeated self-isolation for long periods of time.

Students also suggested this resulted from the perception of being at low risk or through general misinformation about health risks. However, there were also many situations where students considered compliance in balance with other needs, such as the availability of external support (to support practicalities such as shopping), professionalism (if they felt non-adherence would put their future career at risk), risk to others (if they felt their overall risk of having COVID-19 at that time was reasonably low), or for mental health support.

Some staff also felt the reasons given for non-compliance were not genuine in some cases, for example blaming non-compliance on mental health difficulties that did not exist.

### 3.4. Supporting Self-Isolation: Factors with the Potential to Help

#### 3.4.1. Self-Isolation Mindset

In several cases, students adopted a positive outlook about self-isolation. These students felt appreciative of their normal, everyday lives, and for some, isolation fostered a sense of community with others in the same situation. Students described using distraction as a primary tool to help cope with being in isolation. Staff noted that it was important for the university to incentivise compliance with self-isolation by providing activities to support students for the full period of isolation.

#### 3.4.2. University Support

For those self-isolating, the university provided a variety of support measures, including: the logistical aspects of self-isolation (such as meal delivery, care packages, and rubbish collection), emotional support (such as general phone or email communication to offer support, or online self-help tools), activities (such as online social events, tiered isolation), and teaching variations (offering flexible options for teaching). In a small number of cases, students felt that this support was not enough or had not been communicated well.

A small number of staff and students also felt the support was inequitable across faculties and different accommodation locations, with some students receiving greater support than others. This was also visible in how students described their knowledge and experiences of the support available, with some receiving additional support and others saying that receiving this would have been useful.

Staff also described the increased workload and pressures associated with trying to sufficiently provide this range of additional support services to students. Despite support being available, some staff reported low student uptake of the supports offered, in particular the services designed to support student mental health.

#### 3.4.3. Social Support

As a final consideration, peer support was seen by students and staff as being extremely valuable during the pandemic, for both emotional wellbeing and practical support through self-isolation. Both staff and students recognised the need for increased social support for first year and international students, on the basis that their social support networks were likely impeded because restriction measures limited opportunities to build friendships. There was a predominantly positive view towards self-isolating in a group compared to isolating alone, as many students and staff felt that this social support made a significant difference to wellbeing. However, this also came with its own challenges, particularly if there were disagreements, or extended periods of self-isolation due to large numbers of students in a household who may potentially test positive.

## 4. Discussion

The current study provides a qualitative exploration of university students’ and staffs’ experiences of the COVID-19 pandemic, with a focus on the impact of self-isolation. Although the UK government’s roadmap [24] provides a positive route out of national restrictive measures, rising rates of new COVID-19 variants, particularly amongst university students who remain largely unvaccinated, may continue to warrant containment behaviours. We present learnings and potential long-term implications that can be gleaned from the experiences of both students and staff, representing a sector of the current and future workforce.

Students and staff reported initial difficulties adapting to the imposed restrictions and shift to online education. Students, particularly those living in halls, described feeling as though they were trapped in prolonged periods of isolation, given spikes in infection rates within the student body, in tandem with strict national lockdown measures. This posed a significant challenge to students’ expectations of university life, with both students and staff noting that the diminished opportunity to socialise meant many new students were missing out on vital formative experiences. The long-term impact of such experiences should not be overlooked; studies demonstrate that relationships with peers and a sense of university belonging significantly impact students’ social, psychological, and academic outcomes [25,26]. Our findings demonstrate that first year students and international students were particularly impacted by these periods of isolation and lockdown, struggling to develop friendships and social networks as would normally be expected. Although it is unclear whether we will see further periods of enforced isolation following testing, universities must consider the impact that over a year of engaging in stringent protective behaviours has had on student wellbeing, offering opportunities to foster connectedness for students who might have missed them these during the pandemic.

Rapid changes to the modality of teaching provision, i.e., moving from face-to-face to online teaching, were challenging for both students and staff alike. For students, self-isolation was particularly disruptive to their studies and led to concern about the impact of isolation on their learning outcomes. Concern was also noted in relation to general educational outcomes, with some students feeling they had been disadvantaged by this shift. To date, evidence on learning outcomes from online teaching has been mixed, with some studies reporting comparable outcomes to face-to-face teaching [27] and others concluding that online learning leads to worsened outcomes [28]. Both students and staff noted concerns about the impact of online learning on educational outcomes. Monitoring the impact that this year of flexible learning has had on students’ attainment, whilst considering mechanisms to enhance learning outcomes in those affected, will be invaluable. Assessing for professional or educational disadvantages to this model of learning will be particularly important as students transition into the workforce. Similarly, as universities continue to navigate new formats for education delivery, the impact of this delivery on staff who might already face high workloads should be assessed, with appropriate supports put in place to support staff wellbeing.

The emotional impact of self-isolation was multifaceted and diverse, with some students coping well and others experiencing deleterious effects on their mood. For some, self-isolation also fostered a sense of positivity and appreciation of their daily lives post-isolation. In line with these findings, early qualitative work suggests the pandemic has fostered opportunities for appreciation and gratitude by considering one’s personal experience in comparison to others who have been adversely affected (i.e., relative fortune) and has increased time spent with family and engaging in new hobbies or activities [29]. However, burgeoning research has reported the negative impacts of the pandemic and national lockdowns on students’ ability to regulate their attention and motivation levels [30] and to develop studying networks [31]. This was particularly true within our sample, with students reporting reduced motivation to engage with their academic work during isolation. Van Der Feltz-Cornelis and colleagues found that social-isolation was a significant predictor of vulnerability to psychological distress in both students and staff members [15], with additional research reporting increases in self-reported stress, anxiety, loneliness, and depressive symptoms [32,33,34] during the pandemic more broadly. Our findings suggest that environment during isolation is a significant contributor to wellbeing, with those isolating with others seeming to fare better. Similarly, Worsley et al. found that student accommodation offers the opportunity to cultivate a sense of community, with accommodation-based pastoral staff providing an important source of support for general student wellbeing and mental health concerns [35]. This echoes our finding that for some, isolation fostered a sense of community with others who were also isolating. However, for others, feelings of boredom, loneliness, and a lack of separation between work and personal space likely contributed to downturns in their mood. Importantly, both access to greenery or green space and the ability to be physically active offer protective mechanisms against the negative mental and physical impacts of isolation [36,37]. Although we may see a return to pre-pandemic levels of anxiety and depression, this pandemic has highlighted the impact that personal environment and accommodation can have on mental health. Looking forward, as many workplaces and academic environments begin to embrace a work from home culture, consideration for the potential negative impacts on social network building, a lack of division of personal and professional space, and an increased risk of loneliness should be considered.

Despite negative media attention, university students demonstrate high rates of adherence to self-isolation following testing [9]. Students in this study described being mostly adherent, although multiple factors appeared to influence adherence to self-isolation. Students frequently reported pro-social factors as positive influencers of adherence (e.g., moral obligation). Recent research suggests a drive to socialise also impacts adherence to COVID-19 protective behaviours [12]. Indeed, both staff and students viewed peer support as being instrumental during the pandemic, bolstering emotional wellbeing and providing practical support through periods of self-isolation. Students and staff noted that communication factors, such as confusing messaging or general misinformation about health risks may negatively impact compliance. Younger adults frequently derive health information from social media platforms [38], however, there is ongoing concern about the spread of misinformation on through these channels [39]. Importantly, government-supported messaging accounted for only 11% of COVID-19 information on YouTube [40]. Ultimately, students appeared to consider adherence to isolation in balance with practical, professional, and mental health factors. These factors indicate that a multi-pronged approach to facilitating adherence in young adults is required. First, staff noted that adherence to isolation could benefit from incentivisation, aligning with government recommendation [41]. Universities should focus on promoting clear and targeted messaging through relevant channels, such as social media, to help combat misinformation and non-compliant behaviour. Novel campaigns targeting pro-social behaviour may reinforce key drivers of student adherence. Consideration should also be given to the future impacts of isolation on students’ professional goals, for example working with students to develop learning plans for practical placements impacted by isolation. Finally, some students felt that support was not clearly sign-posted and differed by department, whilst staff felt that the offered supports were underutilised at times. The discrepancy between perceived availability and uptake of services warrants further exploration to ensure barriers to access are minimized. Beyond self-isolation, the competing demands placed on young adults to manage practical and professional needs with protective behaviours should be recognised, particularly as rates of COVID-19 infection continue to rise nationally.

To date, there has been limited exploration into the impact of the pandemic on university staff. Stadtlander and Sickel conducted interviews with university faculty and found that whilst staff were generally satisfied with their home workplace, they felt more negative about online work and a loss of freedom and independence. The authors noted that some staff experienced an increase in their working hours as a result of the transition to online working, with some feeling overwhelmed by this transition [42]. The current study purposively sampled staff in student-facing roles from a variety of role families to represent the make-up of the university workforce. Our work suggests staff across all role families struggled to adapt. Many staff reported continued contact with students throughout the pandemic, leading to personal concern about contracting COVID-19, particularly when thinking about their own families and caring for vulnerable others. This anxiety occurred despite recognition that the university provided sufficient protective equipment. The need to support students who were self-isolating also presented a significant challenge to staff, who were expected to deal with a range of resulting issues, from mental health difficulties to non-compliance. Some staff noted changes to their mood as a result, with others finding this additional work added substantially to their workload. For many, this new and reactive way of working induced significant stress, meaning that they were unable to direct attention to other aspects of their role during this time. This led some to feel that greater support for staff was required from the university. Although the initial stressors of adaptation have subsided, we may begin to find that there are mental health sequelae of the prolonged state of reactivity and stress induced by the pandemic. We have seen the impact of this reactivity on the mental health of health care workers [43]. In the current study, it was noted that whilst staff felt services were widely available to students, university support programmes were lacking for staff. As we continue to move through the pandemic, care should be taken to monitor staff stress levels and mental wellbeing, ensuring staff specific support services are available and accessible.

### Strengths and Limitations

This study has several strengths and limitations. The interest and uptake of the focus groups and interviews amongst students and staff demonstrates that remotely conducted focus groups are an acceptable and suitable approach for exploring experiences of self-isolation in both university students and staff during the pandemic. Study rigor was strengthened as all focus group data were collected by two researchers. Additionally, whilst analysis was conducted by one researcher, a second was involved in checking of codes to ensure reliability and rigour of the data. The student sample included both home and international students, although the proportion of home students in our sample is slightly higher than the proportion of home student registrations at the university during the 2019–2020 academic year (82.7% versus 67.9%) [44]. Although measures were taken to improve representative sampling, many students had undertaken asymptomatic testing and were actively involved in COVID-19 related research. Most students also reported being compliant with guidelines, thus, the views of those who are disengaged, ambivalent, or largely non-compliant are likely not represented. We also attempted to recruit a minimum of four participants per focus group and to maximise attendance by offering gift vouchers and scheduling focus groups around the academic schedule. However, given the rapidity of data collection and student non-attendance, we hosted a mixture of larger and smaller student focus groups and one individual interview. Despite the discrepancy in group sizes, the authors felt that the data collected from the smaller groups and individual interview were meaningful, informative, and warranted inclusion within the study. Although our recruitment strategies aimed to achieve demographic representativeness, there were more female than male participants in the sample, with this proportion being higher than the estimated proportion of females across higher education in the UK (80.7% versus 57%) [45]. Studies demonstrate that females find coping with social isolation more difficult than their male counterparts [46] and are more likely to engage in protective behaviours [47] and comply with public COVID-19 measures [48]. Given the gender discrepancy of the sample, our findings are likely skewed towards female viewpoints and experiences and should, therefore, be considered within this context. Future research should endeavour to over-recruit male participants to ensure a reflective gender balance is obtained. During the recruitment period, students received a high volume of COVID-19 related university email correspondence. As our recruitment predominantly occurred through email, students may have missed recruitment emails and focus group reminders. To improve attendance, future studies might consider offering greater incentivization and alternative communication strategies, perhaps through social media or text messaging. Additionally, the proportion of undergraduate compared to postgraduate students was slightly higher than that of student registrations (88.56% versus 72.7% undergraduates) [44]. The majority of students lived in off-campus accommodation and, therefore, the experiences of students living in on-campus university halls of residence may be under-represented. Future research should attempt to explore the views of those who are non-compliant with isolation measures, perhaps through recruitment from university disciplinary channels.

This study has important implications; it is one of the first studies to explore the impact of self-isolation on students and staff in British higher education. However, the data should be considered in terms of the context in which they were collected. Focus groups and interviews were conducted during the third national lockdown, during which many students were, in effect, experiencing self-isolation to some degree. Thus, some of the findings may not be specific to containment behaviours, but more reflective of the experience of lockdown measures in general. Groups took place shortly after the winter break, and so attitudes towards testing and uptake of symptomatic testing may be influenced by the fact that these were encouraged before travelling home, and so may not be representative of students’ attitudes during other time-points. Finally, it is worth noting that all students and staff were from a single institution, which experienced a large COVID-19 outbreak at the beginning on the Autumn term, resulting in a large number of students self-isolating and the imposition of stringent local restrictions. However, data were collected during a national lockdown and, thus, likely represent the student and staff experiences of large campus-based universities in England.

## 5. Conclusions

The COVID-19 pandemic has significantly impacted students and staff, who were required to make rapid adjustments to novel learning environments. Although adherence to containment behaviours appears high amongst students, current and future mental health implications of isolation should be considered. Ensuring adequate practical, social, and emotional support for both students and staff will be paramount moving forward, given that containment behaviours may be required until high rates of vaccination are achieved in this population. Greater support should also be provided for staff during transitional periods, with ongoing monitoring of workforce stress levels warranted.

## Figures and Tables

**Table 1:** Sample characteristics of students.

Characteristics (*n* = 52)	Mean (Range)/*n* (%)
Age *	19.7 (18–33) years
Gender	
Male	10 (19.2)
Female	42 (80.8)
Student status	
Home students	43 (82.7)
International students	9 (17.3)
Accommodation type	
On-campus	5 (9.62)
Off-campus	42 (80.8)
Unclear/missing	5 (9.6)
Academic Status	
Undergraduate	46 (88.6)
Postgraduate	5 (9.6)
Unclear/missing	1 (1.9)
Year of study	
Undergraduate first year	12 (23.1)
Undergraduate second year	8 (15.4)
Undergraduate third year	17 (32.7)
Undergraduate fourth year	1 (1.9)
Postgraduate	4 (7.7)
Missing	10 (19.2)
Testing status	
Not tested	3 (5.8)
Received any test	49 (94.2)

* 5 missing data.

**Table 2:** Sample characteristics of staff.

Characteristic (*n* = 26)	*n* (%)
Gender	
Male	7 (26.9)
Female	19 (73.1)
Role category	
Student health and wellbeing	4 (15.4)
Accommodation support and residential experience	11 (42.3)
Teaching and academic support	4 (15.4)
COVID testing operations	2 (7.7)
Student experience and pastoral care	5 (19.2)

**Table 3:** Examples of key themes, subthemes, frequency, and their representative quotes.

Theme 1: Adaptation during the Pandemic	
A new normal	I think that just prolonged isolation in general, the whole thing being in lockdown since March and whatnot since March, but just not really going out and socialising has changed me in a way that made me really comfortable with solitude and a little bit distressed when I’m outside. FG7, S1 I think it’s getting back to normal now because, I don’t know, I think at first it was a bit weird again to see each other. It almost felt like we were doing something wrong, but we’ve got over that stage and it’s far more natural now to just, you know, chill and chat. FG7, S2
Challenges of adapting during COVID-19	We were wondering whether our marks would get negatively affected from not being legally allowed to go to those things. FG1, S8 Most students I know do think that we are definitely at a disadvantage because it’s just a completely different environment to be learning in really. FG6, S3 And then we had to do the remote teaching including you know, we’re just different because we’ve got this dual cohort not that started for the first time last year so we had 150 students start in April during lockdown, so we were remote teaching them without them ever having been on campus. And then they came on campus in July to catch up on the practical teaching so they’re just completely different to the normal university students. Staff interview 18 I just think there’s a huge expectation about you’re going to come to university and there’s going to be parties and you’re going to make loads of friends. And even though they came to university understanding that they weren’t going to get that, I guess it was kind of inevitable that to repress and suppress those expectations, was always going to be verging on impossible. Staff interview 14 There’s one concern, particularly for the practical people on the ground which is obviously their fear of contracting the virus, you know and the sense that the University is putting them in jeopardy, and is not sufficiently supporting the health and wellbeing, through a health and safety prospective. Staff interview 2 It was quite frightening at first because you just feel like you’re forever firefighting, because it was en-masse people were told to, like, you know, pack up and all move down to our hall, I mean, we had no masks, you know, there were people who were frightened. Staff interview 5
**Theme 2: The Practical, Environmental and Emotional Challenges of Self-Isolating**	
Self-isolation environment	The thing that I couldn’t even cope with for that 24 h I was there, was doing everything in one room, like exercise, uni work, sleeping, relaxing, reading, eating, all of it in one single room. I need separation, I need to be able to step away from things. FG1, S2 I was in a house with 8 so there was 8 of us there and we could all do things like, I don’t know, we sat down and watched some TV together or we watched a movie or we played a game. I think just having a lot of people there helped to keep me distracted and not get as worried about the whole situation. FG27, S3 I’ve had quite a few conversations with international students more recently where I’ve had a student that recently said “I’ve not been in the presence of another human being since September” and they’ve been in Nottingham. They’ve moved away from their home country to come here and not had virtually any contact with anybody at all and have been self-isolating by their own choice. And again, that is a real issue because they literally have no support network here whatsoever. Staff interview 14
Emotional effects of self-isolation	To me it didn’t really make much of a difference because we were already in lockdown but yeah, I think some of my friends found it a bit harder because they had other friends outside the house that they enjoyed seeing, but personally I was fine with it. FG17, S3 I found it hard to concentrate on my studies because I was stuck in my room all day, I just found the motivation side really tricky. FG1, S7 When I had to self-isolate it was a bit like, for me I was like missing out on the social part of it, which affected my mood a bit and like my motivation to work was really affected at the time. FG17, S2 I was the first person in my household to actually test positive for Covid and have symptoms I felt quite guilty that the rest of my house had to isolate. FG5, S2
How self-isolation affects university service provision	Some halls we were at 70% students in isolation. So, the biggest issue was how we serviced these students, how we got food to these students from a catering perspective, dealing with um, you know dietary requirements and getting all of that information through. Um, cause we, we just didn’t have a system that could cope with that to be fair. Staff interview 11 I think it would be nice if the staff had something, you know, more than just a letter to say, you know, this week we’re going to say thank you to other people, you know, the gardeners for making the area look nice, you know, it should be that the staff can be able to talk to somebody and there’s nobody to talk to private and confidential. The staff have got problems at home as well, you know, they’re coming into work with families that’s ill and then we’ve got schooling problems. And I don’t think the university are looking at that. Staff interview 12 It’s been so difficult this year to plan any work, I definitely feel that the majority of my work has been reactive. Staff interview 7
**Theme 3: Social Factors and Their Impact on COVID-19 Testing, Vaccinations, and Self-Isolation Adherence**	
Testing: social factors, barriers and enablers	I’d say that one of the best parts that the university—well, one of the best things the university has done was that big mass testing that they did when everyone was leaving. When I was speaking to some of my friends at other universities, they didn’t actually have that, which I was kind of surprised about because I thought it would be every university. It was organised very well, it was carried out super efficiently. FG27, S4 I was definitely happy to have it cause it meant I could come home and not feel guilty for being ‘a spreader’. FG3, S1 I think um, that a need to avoid self-isolating has led to people not following the guidance, and has actually led to people harassing other students. Um, because someone goes and has symptoms and gets a test, it then means everyone’s got to self-isolate, so I think there’s been pressure to either for people not to test, um, and for people to, or not to share that they’ve tested. Staff interview 20
Compliance with self-isolation	I think also people with um like mental illness, or, not necessarily illness but just, um, er struggling with their, with poor mental health. And I think if, if obviously it’s better to probably, I mean I know it’s technically breaking the law but it’s probably better to meet up with someone when you’re in self-isolation if you’re having for example suicidal thoughts. FG4, S3 I think where other people before they thought they might have to go into isolation they quickly did a food shop, we were able to not do this because we all had friends that weren’t in isolation and didn’t have any symptoms, so they could bring us food to last us until we could get a delivery slot. FG5, S1 I think not all students take it equally seriously, and I think that’s the main thing is that a lot of them just kind of don’t see the point, and there’s also a lot of misleading information that young people are less likely to get covid badly. FG3, S1 They absolutely weren’t happy and I don’t think they understood why they should be self-isolating. But on the other hand we have students who always follow the rules and do as they’re told. Staff interview 15 Almost 100% of those students who were found to repeatedly breach Covid rules, and their letters of appeals to the registrar, cited severe mental health problems as a reason to not adhere to the rules... and actually we found that almost 100% of the students didn’t have significant mental health problems. Staff interview 17
**Theme 4: Supporting Self-Isolation: Factors with the Potential to Help**	
Self-isolation mind-set	It was quite nice and it very much lifted the mood of kind of—because before isolation it’s, kind of, you don’t really—you kind of just take it for granted because, as I said, I wasn’t going to the university really at all, so actually being able to isolate and then come out made me feel a lot better about being out. FG5, S4 I think when they first got there, whole blocks were having to isolate. It was almost excitement, ‘ohh, we’ll do it together’, do you know what I mean? Staff interview 22 I think after a couple of days I started coping a bit better because I had ways to distract myself, occupy myself with work or other things. So it got a lot easier throughout it. FG27, S1 I think that what the university wants to say is that it’s important to self isolate, so, and it is also an incentive for them to self isolate for all the period, not only for the few days and maybe you’ve got your fever is just disappeared and you’re young and so you want to go out. Giving them something to do for seven days, ten days makes them maybe feel that it’s really important to do that and it’s also an incentive. Staff interview 16
University support	I was made aware of the options that were given because I remember when the first lockdown started, we received loads of emails from the university telling us about options if we wanted to get in contact with anyone about mental wellbeing, or any kind of support from either the university or your own course or department, which I found very helpful but personally I didn’t use any of those. FG14, S2 I really liked the university doing was they had a lot of sessions about kind of mental health, but one of the problems is you had to search for them, or they were hidden in the, halfway through an email, so it wasn’t that the university hadn’t provided it, it was that kind of, it was there but you have to go and find the necessary links and kind of do it. FG2, S7 I mean for the students in Halls it’s better because they should get their food deliveries every day and they’ve got the duty hall managers that will be checking in and checking they’re all right. But for students who are living in their own accommodation, they’re going to become very isolated and I mean there’s no guarantee that they’ve got anybody than can get food to them. Staff interview 9 I mean generally there would have been face-to-face contact. This year we’ve tried to do that via Teams, but students haven’t particularly engaged in that, to be fair, it’s been, it has been quite difficult in that respect. Staff interview 11
Social support	I cope with that a lot of the time by socialising and seeing people a lot, um so I was lucky my girlfriend came and spoke to me through my window. FG1, S7 What made it a lot easier for me was like a lot of my friends knew I was in isolation so they’d call me very often. But I guess if it was a day where I didn’t get like a phone call or one of them was busy or something like that, because I wasn’t living with my family or housemates or anything, I’d just spend like the whole day by myself. FG27, S2 I think being part of the community where they kind of make it easier and more enjoyable to stay at home, I think that definitely, definitely made it easier to stay at home and adhere to it. FG4, S3 I think that’s been especially hard on the first year students, especially first year international students where they don’t have a support network. Staff interview 14 I think the most vulnerable were also the first year students so they were here for the first time. They didn’t know anybody and it’s tougher for them so probably they also tend to approach the university more because they don’t know how to ask for help and support. Staff interview 16 I think someone said earlier, but it was a bit of a, like being in first year and with like um, seven people you’d sort of just met, um there was a bit of a like blame game going on where it was like, oh who got the test, who meant we had to isolate, like that caused a bit of tension, and so yeah that was sort of a challenge like to get through like, when you’ve only known these people y’know a few weeks. FG2, S6

## Data Availability

The data presented in this study are available on request from the corresponding author. The data are not publicly available due to risk of participant identification.

## Supplementary Materials

The following are available online at https://www.mdpi.com/article/10.3390/ijerph182010675/s1, File S1: Study themes and subthemes with supporting quotations, File S2: Consolidated criteria for reporting qualitative studies (COREQ), File S3: Student and staff interview Guides. Supplementary File S4. Thematic map illustrating the relationships between key themes and subthemes.
