# Supplementary material for: Impacts of the COVID-19 Pandemic and Self-Isolation on Students and Staff in Higher Education: A Qualitative Study
Source: Int J Environ Res Public Health. 2021 Oct 12;18(20):10675. doi: 10.3390/ijerph182010675 (PMC8535702; doi:10.3390/ijerph182010675)

Supplementary File 1. Study themes and subthemes with supporting quotations.

Theme/Subtheme	Supporting Quotations
**Theme 1: Adaptation during the pandemic**	
A new normal	I think that just prolonged isolation in general, the whole thing being in lockdown since March and whatnot since March, but just not really going out and socialising has changed me in a way that made me really comfortable with solitude and a little bit distressed when I'm outside. FG7, S1<br><br>I think it's getting back to normal now because, I don't know, I think at first it was a bit weird again to see each other. It almost felt like we were doing something wrong, but we've got over that stage and it's far more natural now to just, you know, chill and chat. FG7, S2
Challenges of adapting during COVID-19	We were wondering whether our marks would get negatively affected from not being legally allowed to go to those things. FG1, S8<br><br>I know quite a lot of other people who found similar, and dropped behind on lectures over their isolation period. Because they just found it so much harder to get work done. FG1, S7<br><br>I think things like the grace periods and giving a bit more relaxation on deadlines and uni work, I think it takes the stress away from um uni work so then you can just focus on for example, doing what you need to do to look after your mental health, so that you can actually adhere to the self-isolation without actually stressing about your uni life cause your able to just look after yourself. FG4, S3<br><br>I can imagine it being quite difficult, especially when you haven't been to uni at all and this is your first year to then having to figure out not just how to do university learning, but university learning online, because the process of lecture engagements and separate to lectures is very different for a lot of people and that was quite difficult for me having to figure out. FG6, S6<br><br>most students I know do think that we are definitely at a disadvantage because it's just a completely different environment to be learning in really. FG6, S3<br><br>I guess for first years at least I, I knew some people in first year, it was their first time away from home, um, so I can see why they might have felt, felt a bit daunted by not knowing anyone around them or things like that. FG1, S8<br><br>I think what I've heard several times is 'this should have been special, these are my special years', OK. Now if you manage young people's expectations like that, we know in counselling people come to university thinking, being told it's going to be amazing, it's going to be the best years of your life – and then you lock them up in a tiny little room with a desk and a bed and some cruddy meals – they're not going to feel OK about it. Staff interview 13<br><br>I just think there's a huge expectation about you're going to come to university and there's going to be parties and you're going to make loads of friends. And even though they came to university understanding that they weren't going to get that, I guess it was kind of inevitable that to repress and suppress those expectations, was always going to be verging on impossible. Staff interview 14<br><br>There's one concern, particularly for the practical people on the ground which is obviously their fear of contracting the virus, you know and the sense that the University is putting

	them in jeopardy, and is not sufficiently supporting the health and wellbeing, through a health and safety prospective. Staff interview 2<br><br>So firstly, our housekeeping staff are very anxious because they obviously have people at home, they have family, they may be caring for people who are vulnerable, so for them to start entering areas where they know people are self-isolating and a person has tested positive for Covid-19, they are very anxious into catching it, even though they are wearing all the PPE that they need to wear. Staff interview 25<br><br>And then we had to do the remote teaching including you know, we're just different because we've got this dual cohort not that started for the first time last year so we had 150 students start in April during lockdown, so we were remote teaching them without them ever having been on campus. And then they came on campus in July to catch up on the practical teaching so they're just completely different to the normal university students. Staff interview 18<br><br>It was quite frightening at first because you just feel like you're forever firefighting, because it was en-masse people were told to, like, you know, pack up and all move down to our hall, I mean, we had no masks, you know, there were people who were frightened. Staff interview 5

Theme/Subtheme	Supporting Quotations
**Theme 2: The practical, environmental and emotional challenges of self-isolating**	
Self-isolation environment	So this small environment that you're stuck in was turn, started turning like in to a very negative environment because you had all your rubbish in one place, you couldn't take your bins out, and it just made the whole experience much more difficult in general. FG1, S4<br><br>I share a room with my sister, so I kind of didn't have any of my own space, and so I think that was quite difficult in the sense that I couldn't leave the house to get some me time. Um, but yeah, I think that was probably one of, one of the, one of the challenges of isolation in a group where you are in a house with other people. FG4, S3<br><br>the thing that I couldn't even cope with for that 24 hours I was there, was doing everything in one room, like exercise, uni work, sleeping, relaxing, reading, eating, all of it in one single room. I need separation, I need to be able to step away from things. FG1, S2<br><br>I was in a house with 8 so there was 8 of us there and we could all do things like, I don't know, we sat down and watched some TV together or we watched a movie or we played a game. I think just having a lot of people there helped to keep me distracted and not get as worried about the whole situation.<br>FG27, S3<br><br>it is very good for your mental health to have people you can be with and talk to and people that you know around you because, yeah, you do just share the same kitchen, you can't clean everything perfectly every time you use it, so isolating as a whole house was definitely better than isolating in my room or telling the person who tested positive to just never come out of their room, because it's not really fair on them as well to say 'you can't leave your room because we don't want Covid'. FG5, S4

	The hall I work at was described as 'prison halls' because there were that many students isolating and they weren't able to leave their rooms. Staff interview 25<br><br>We then had discussions about this following student feedback and following some of the issues that were iden- you know, complaints I suppose, and trying to make it more bearable for them to stay in the rooms. That's when we then spoke to Public Health England and looked at what we could do, can we get them some outdoor space, and could we allow some of them to come to the dining room, so at least they can come out of their room, go and get some space, go and get some air, eat in the dining room. Cause obviously the problem with eating everything in your room, you then get associated issues with rubbish building up in the rooms, smells in rooms, and the creation of a, you know, not a very nice environment to be living in 24 hours a day, cause you're talking about a four-by-four room here, it's not huge. Staff interview 11<br><br>I'm fortunate, I've got a garden, so I can go and stand outside if I want some fresh air, whereas some of these students haven't got that, they're living in these high rise flats and they can't go anywhere apart from that floor and I can imagine that's difficult. Staff interview 23<br><br>I've had quite a few conversations with international students more recently where I've had a student that recently said "I've not been in the presence of another human being since September" and they've been in Nottingham. They've moved away from their home country to come here and not had virtually any contact with anybody at all and have been self isolating by their own choice. And again, that is a real issue because they literally have no support network here whatsoever. Staff interview 14
Emotional effects of self-isolation	To me it didn't really make much of a difference because we were already in lockdown but yeah, I think some of my friends found it a bit harder because they had other friends outside the house that they enjoyed seeing, but personally I was fine with it. FG17, S3<br><br>I found it hard to concentrate on my studies because I was stuck in my room all day, I just found the motivation side really tricky. FG1, S7<br><br>When I had to self-isolate it was a bit like, for me I was like missing out on the social part of it, which affected my mood a bit and like my motivation to work was really affected at the time. FG17, S2<br><br>Yeah, my, my mood, my mum's mood, everyone's mood just kind of dropped. And we're normally, we normally make an effort to be kind of, get up and go. FG3, S1<br><br>So I didn't leave my flat at all for the entirety of the two weeks, which was very hard, is very hard on your brain, because I like to walk. FG5, S4<br><br>I feel like first year just – it's like the year where you meet people and you make all your friends – and even when they weren't in isolation, they were barely able to meet people and then they were all just in and out of isolation all the time, especially like obviously in some halls you're in a whole block and you all have to go in isolation at the same time so, like, if one person out of that 20 test positive then the whole block is in isolation. FG5, S3<br><br>I was the first person in my household to actually test positive for Covid and have symptoms I felt quite guilty that the rest of my house had to isolate. FG5, S2

How self-isolation affects university service provision	Some halls we were at 70% students in isolation. So, the biggest issue was how we serviced these students, how we got food to these students from a catering perspective, dealing with um, you know dietary requirements and getting all of that information through. Um, cause we, we just didn't have a system that could cope with that to be fair. Staff interview 11<br><br>So I think in my team we had about fifty calls between the four of us in my team to do. So I think I might have done about twenty of those, and again that was a mixture of people that wanted, had practical questions, or that people were upset about paying accommodation or things that had happened in halls that they were not happy about – fees, all sorts of things like that. But there were lots of other questions in there from students that were due to go on a year abroad about "What's going to happen with my course? No one's given me an indication. I've applied for ECs. I've not heard anything". So again it was more kind of practical questions that were generated. Staff interview 14<br><br>I think it would be nice if the staff had something, you know, more than just a letter to say, you know, this week we're going to say thank you to other people, you know, the gardeners for making the area look nice, you know, it should be that the staff can be able to talk to somebody and there's nobody to talk to private and confidential. The staff have got problems at home as well, you know, they're coming into work with families that's ill and then we've got schooling problems. And I don't think the university are looking at that. Staff interview 12<br><br>You know and we tried very hard to get the hall treated as a household, but Public Health England would not allow us to do that. Which would have made it a lot easier for us to manage, you know and control that. Staff interview 11<br><br>I think staggered returns would have been better, you know, to be able to test the water, see, I think, but I think it was made impossible for us because the government were just not going to help us, and I think that was a real shame and I think we were put in a really difficult position. Staff interview 20<br><br>It's been so difficult this year to plan any work, I definitely feel that the majority of my work has been reactive. Staff interview 7

**Theme/Subtheme**	**Supporting Quotations**
**Theme 3: Social factors and their impact on COVID-19 testing, vaccinations and self-isolation adherence**	
Testing: social factors, barriers and enablers	I'm glad the university provided asymptomatic testing, just to have peace of mind to know that we weren't bringing anything back to our families was quite supportive. FG17, S5<br><br>I'd say that one of the best parts that the university – well, one of the best things the university has done was that big mass testing that they did when everyone was leaving. When I was speaking to some of my friends at other universities, they didn't actually have that, which I was kind of surprised about because I thought it would be every university. It was organised very well, it was carried out super efficiently. FG27, S4<br><br>I was definitely happy to have it cause it meant I could come home and not feel guilty for being 'a spreader'. FG3, S1<br><br>Yeah, when the students have to go home they had to have a test to say that they was negative before they could travel. They was ignoring having that test, they were just leaving and not telling us they was leaving so they could avoid having the test. Staff interview 12

	I've heard of other students who have proactively decided not to get tested because they don't want to know, and have decided that they're going to go home because they think that a positive test might be coming, if their housemate's gone. And before they've been officially told to go home and isolate they've already decided they're going to go on the train and go home because they don't want to spend that isolation in a room on their own. Staff interview 14<br><br>I think um, that a need to avoid self-isolating has led to people not following the guidance, and has actually led to people harassing other students. Um, because someone goes and has symptoms and gets a test, it then means everyone's got to self-isolate, so I think there's been pressure to either for people not to test, um, and for people to, or not to share that they've tested. Staff interview 20<br><br>So it's kind of unnecessary for students who live outside of the city to just go all the way to the campus to take a test. One thing they can do is just offer different test centres in different parts of Nottingham. FG14, S2<br><br>Food is an excellent bribery for student, do the, stick a thing up your nose and get a 15% off pizza voucher, that, that sort of thing. Um, you know it's a step up from going to the dentist and get a lollipop, but it works. FG3, S1<br><br>Placements are really important for you and it's part of your development. In fact it's probably one of the biggest reasons why you've come to university, to get that sort of experience. If you want to partake in that, these are the, you know these are the hurdles you've got to get through and I think the schools have been quite good at enforcing that with their, enforcing that message which has driven people getting testing and repeat testing. Staff interview 4
Compliance with self-isolation	We kind of like separated as a house, so like we isolated from the person like, the person who was positive, um and so I could, I still isolated but knowing I was like, I didn't because I tested negative, and I think it was like day 9, I think, like I went for a run, so like, yeah that's not allowed, but I didn't come into contact with anyone, so like morally in my head I was okay by doing that. FG1, S3<br><br>I think people who'd had to isolate before or who'd tested positive like before, um, were like less likely to you know comply with the rules, um because they obviously thought oh like they're less likely to spread it cause they've already had it or whatever. FG2, S6<br><br>Part of it is you know, certainly my conversations with the students who were being more careful, always tended to be students who had family members who were vulnerable. Staff interview 20<br><br>There's quite a range of adherence! It's quite variable I think. I think there are different groups – I couldn't give you figures or numbers for this – but there'll be the group of compliant students who want to do the right thing who have a very strong sense of responsibility to their own families, to their peers and to the organisation and they take it very seriously, to the extent that when they see some of their peers breaking the rules it is distressing for them. Then you've got your group who are sort of trying to be good and trying to be young and free and have a good time at the same time and you'll have a bit of rule bending. Staff interview 13

I can imagine that if the pandemic had happened when I was in my first year, I would have found it really hard to not still go and see some friends in other halls of residence. So I think it does make sense because they are probably more willing to break the rules just to socialise a bit more. FG14, S1

I think also people with um like mental illness, or, not necessarily illness but just, um, er struggling with their, with poor mental health. And I think if, if obviously it's better to probably, I mean I know it's technically breaking the law but it's probably better to meet up with someone when you're in self-isolation if you're having for example suicidal thoughts. FG4, S3

And very early on, you know going, you know, "here's some Amazon vouchers, buy a film", "here's some pizzas, stay indoors". You know I think making it more difficult to break than easier to break it is important. So, making people feel that it's, that actually self-isolation is the right thing to do. And actually, you've made it easy because you've brought them food that is good quality food, and you've given them high quality internet, so they've got that and you've given them opportunity to engage in some form of outdoor exercise. Like kind of reducing their reasons for wanting to break that self-isolation, on a practical level, I think are important. Staff interview 2

I think my house and I were able to follow the rules quite well because we were able to get food delivered, but I think if you weren't able to get food delivered for any reason I think you would definitely have to leave and go to the shops unless you had friends that could bring it to you. FG17, S3

I think where other people before they thought they might have to go into isolation they quickly did a food shop, we were able to not do this because we all had friends that weren't in isolation and didn't have any symptoms, so they could bring us food to last us until we could get a delivery slot. FG5, S1

Firstly because of being a medical student, if I have a positive result and I go against that, that is real fitness to practice. So again, that's just unacceptable so I have no problems adhering to isolation whatsoever. FG7, S1

I think a lot of people come out of their isolation period a few days early, they get sort of bored of the isolation, feel that they're safe and don't have symptoms and then come out of their isolation period early. FG1, S7

I think not all students take it equally seriously, and I think that's the main thing is that a lot of them just kind of don't see the point, and there's also a lot of misleading information that young people are less likely to get covid badly. FG3, S1

Some people got called by the NHS track and trace, like, every day in my house, like, a few of us, like, a couple of us got called one and a half weeks into our two week isolation, like, there seemed to be no rhyme or reason to it, but we were getting told, like, different things over text and call about how long we had to isolate for. FG5, S1

They absolutely weren't happy and I don't think they understood why they should be self-isolating. But on the other hand we have students who always follow the rules and do as they're told. Staff interview 15

	I live in a studio but on the same floor there's like a flat next to me which are like my support bubble kind of friends and then some of them also had to self-isolate but then me being alone in a studio, basically I kind of had a fear of missing out as well because some of them didn't have to self-isolate. When I had to self-isolate it was a bit like, for me I was like missing out on the social part of it, which affected my mood a bit and like my motivation to work was really affected at the time. FG17, S2<br><br>Almost 100% of those students who were found to repeatedly breach Covid rules, and their letters of appeals to the registrar, cited severe mental health problems as a reason to not adhere to the rules... and actually we found that almost 100% of the students didn't have significant mental health problems. Staff interview 17

**Theme/Subtheme**	**Supporting Quotations**
**Theme 4: Supporting self-isolation: factors with the potential to help**	
Self-isolation mind-set	When we all got together, we've all isolated at some point or other um, so that was, that was very different feeling of meeting up. So, I think going through that period of self-isolation does change how you see sort of the value of friendships and meeting up with people. FG3, S1<br><br>It was quite nice and it very much lifted the mood of kind of – because before isolation it's, kind of, you don't really – you kind of just take it for granted because, as I said, I wasn't going to the university really at all, so actually being able to isolate and then come out made me feel a lot better about being out. FG5, S4<br><br>I think when they first got there, whole blocks were having to isolate. It was almost excitement, 'ohh, we'll do it together', do you know what I mean? Staff interview 22<br><br>Generally, there was quite a collegiate approach, most students were getting on with it and I think there was a bit of a 'we're all in this together' type attitude. Staff interview 24<br><br>Another thing that helped me is having a laptop so I can play games or watch Netflix or do work or any other things, I can do that. Yeah, that's pretty much what helped me. FG14, S3<br><br>I had to self-isolate I was in a house with 8 so there was 8 of us there and we could all do things like, I don't know, we sat down and watched some TV together or we watched a movie or we played a game. I think just having a lot of people there helped to keep me distracted and not get as worried about the whole situation. FG17, S3<br><br>I think after a couple of days I started coping a bit better because I had ways to distract myself, occupy myself with work or other things. So it got a lot easier throughout it. FG27, S1<br><br>I think that what the university wants to say is that it's important to self isolate, so, and it is also an incentive for them to self isolate for all the period, not only for the few days and maybe you've got your fever is just disappeared and you're young and so you want to go out. Giving them something to do for seven days, ten days makes them maybe feel that it's really important to do that and it's also an incentive. Staff interview 16<br><br>So if one of them had it, the whole block was isolating. So they'd respond to that quite well because they're just partying the whole time because they're all isolating together. Staff interview 22

	I was made aware of the options that were given because I remember when the first lockdown started, we received loads of emails from the university telling us about options if we wanted to get in contact with anyone about mental wellbeing, or any kind of support from either the university or your own course or department, which I found very helpful but personal I didn’t use any of those. FG14, S2<br><br>I really liked the university doing was they had a lot of sessions about kind of mental health, but one of the problems is you had to search for them, or they were hidden in the, halfway through an email, so it wasn't that the university hadn't provided it, it was that kind of, it was there but you have to go and find the necessary links and kind of do it. FG2, S7<br><br>So in that sense we were really lucky but speaking to some of my friends in other third party accommodation, they had a lot more difficulty in securing food and there was a lot of uncertainty with that. I think one major thing that would have helped is having accessibility to that without the extra hassle. So the university perhaps organising some kind of service that they could use would have been helpful for them, but for me, from my personal perspective, that was fine. FG27, S1<br><br>If there was some way the university could at least check in with students who were self-isolating, find out whether they, you know, were being looked after by their accommodation, whether their psychological welfare was okay, um, whether they were getting out to do exercise, maybe some like small space exercise videos or something. FG3, S1
University support	I contacted my tutor as well and she offered to deliver food to me as well, so I think certain people in the university really were a great support system. FG5, S2<br><br>my module convener and my tutor, they did email to see if I was OK and if I need anything, but then, like, that was it. And I feel like maybe they could follow up just to check because, like, I know, like, for me personally, I'm not really the type of person to go and ask for help if I did need it. FG6, S1<br><br>Because I know they sent out like care packages, which was really nice. There was like a loaf of bread and like hand sanitizer and they had some other stuff, which was really nice. But that was only once. FG8, S1<br><br>I hope that the meals being delivered to them helps them. Other than that, the onus is very much on them to reach out for anything else, but I would hope that the things that we’re doing operationally do help them get through that isolation period. Staff interview 6<br><br>I mean for the students in Halls it’s better because they should get their food deliveries every day and they’ve got the duty hall managers that will be checking in and checking they’re all right. But for students who are living in their own accommodation, they’re going to become very isolated and I mean there’s no guarantee that they’ve got anybody than can get food to them. Staff interview 9<br><br>They look after the accommodation, and they will organise food parcels to be delivered, and anything else that they need, if they need any medications picking up, they organise that. They’ve been absolutely fantastic. Staff interview 1

	you know it’s not just offering academic support it’s, you know they put on social events for them as well which is really, really good. Staff interview 1<br><br>the university has tried to put those mechanisms in place where they’ve got regular contact with people and I think those students that were self-isolating had like access to some pre-recorded information that they could watch maybe at times when they didn’t have their lectures so they’d got that interaction. And they also were supported with sort of food deliveries and then obviously the exercise side of it. Staff interview 10<br><br>So we did try listening to the students, and we did try to do what we could do to make it a little bit more bearable for them but, you know, at the end of the day, it is what it is. Staff interview 11<br><br>When they first come into hall they have a get together, they have a few quizzes and that and they say, you know, I'm the tutor if you need anything, you know, they're here for their welfare. But I think we've had a big lack of that this year, we haven't had that, we haven't had the care that’s needed. Staff interview 12<br><br>an awful lot was done, you know, they put up these marquees I think outside the halls and it just went horribly wrong when they turned into parties and lots of rule breaking! So I would argue – and this is no criticism – I think everybody – I mean government – I'm not even going there – but certainly the university has done – and universities generally – have worked bloody hard to do what they can. Staff interview 13<br><br>Now my understanding is that if they're in halls, people will, hall staff will go and knock on the door and check on them, they're not just left on their own for ages and ages. I think their experience of that might be a bit more variable. And that’s probably changed during the course of the different lockdowns as well. Staff interview 13<br><br>I mean generally there would have been face-to-face contact. This year we've tried to do that via Teams, but students haven't particularly engaged in that, to be fair, it's been, it has been quite difficult in that respect. Staff interview 11
Social support	I cope with that a lot of the time by socialising and seeing people a lot, um so I was lucky my girlfriend came and spoke to me through my window. FG1, S7<br><br>I’m not trying to be rude but I feel like there’s not much actually the university can do that they can offer to students and what students actually can take from it in terms of during the whole self-isolation period. Obviously it’s being alone and you just want like personal connections, personal contact with people that you keep in touch with. FG14, S2<br><br>I think like having that support as well from like my flatmates definitely made it easier, um than I expected it to be. FG2, S1<br><br>what made it a lot easier for me was like a lot of my friends knew I was in isolation so they’d call me very often. But I guess if it was a day where I didn’t get like a phone call or one of them was busy or something like that, because I wasn’t living with my family or housemates or anything, I’d just spend like the whole day by myself. FG27, S2<br><br>I think being part of the community where they kind of make it easier and more enjoyable to stay at home, I think that definitely, definitely made it easier to stay at home and adhere to it. FG4, S3

	I've just kind of dropped them a line and they've told me when they're coming out of self isolation and that's that. They just, they don't need my support because they've got it already with their peers. Staff interview 18<br><br>We talk about the emotional frame of safety, frame of emotional safety. Which I know is an emotional safety, I think academically is a different thing. But we basically say look there's a scaffold that people have, when you think about the kind of the platform that they stand on, which is, you know you have your friends, you have your family, you have your social structure and you have your academic structure. Staff interview 2<br><br>Most student households are made up of people that are friends, get on well with each other and will support each other. So they're quite supportive environments in which one has to self-isolate. Staff interview 24<br><br>I couldn't imagine being in first year and say if you don't really get on with your flat or not really having such support there, it could have been harder. FG5, S2<br><br>I think that's been especially hard on the first year students, especially first year international students where they don't have a support network. Staff interview 14<br><br>I think the most vulnerable were also the first year students so they were here for the first time. They didn't know anybody and it's tougher for them so probably they also tend to approach the university more because they don't know how to ask for help and support. Staff interview 16<br><br>I think someone said earlier, but it was a bit of a, like being in first year and with like um, seven people you'd sort of just met, um there was a bit of a like blame game going on where it was like, oh who got the test, who meant we had to isolate, like that caused a bit of tension, and so yeah that was sort of a challenge like to get through like, when you've only known these people y'know a few weeks. FG2, S6

EC = Extenuating circumstances

Supplementary table S2. Checklist for adherence to the consolidated criteria for reporting qualitative studies (COREQ)

**Topic and Item No.**	**Guide Questions/Description**	**Response**
	**Domain 1: Research team and reflexivity**	
	Personal Characteristics	
1. Interviewer/facilitator	Which author/s conducted the focus groups?	Morna O'Connor (MO), Sophie Carlisle (SC)
2. Credentials	What were the researcher's credentials? E.g. PhD, MD	MO: PhD<br>SC: MSc
3. Occupation	What was their occupation at the time of the study?	MO: Researcher/ PhD student<br>SC: Researcher/PhD student
4. Gender	Was the researcher male or female?	2 females
5. Experience and training	What experience or training did the researcher have?	MO and SC: Mixed-methods researchers, experienced interviewer, GCP trained
	Relationship with participants	

6. Relationship established	Was a relationship established prior to study commencement?	Researchers met the participants during recruitment
7. Participant knowledge of the interviewer	What did the participants know about the researcher? e.g. personal goals, reasons for doing the research	Participants knew that SC and MO were PhD students and researchers. They knew that both interviewers were affiliated to the participating university.
8. Interviewer characteristics	What characteristics were reported about the interviewer/facilitator? e.g. Bias, assumptions, reasons and interests in the research topic	Participants knew that MO and SC were interested in the experiences of self-isolating students during the COVID-19 pandemic.
**Domain 2: Study design**		
	Theoretical framework	
9. Methodological orientation and Theory	What methodological orientation was stated to underpin the study? e.g. grounded theory, discourse analysis, ethnography, phenomenology, content analysis	Thematic analysis
	Participant selection	
10. Sampling	How were participants selected? e.g. purposive, convenience, consecutive, snowball	Purposive (e.g. for students - self-isolated, COVID-test; for staff – had student facing role)
11. Method of approach	How were participants approached? e.g. face-to-face, telephone, mail, email	Participants were approached and recruited by email
12. Sample size	How many participants were in the study?	52 participants took part in qualitative focus groups, and 26 participants took part in staff interviews
13. Non-participation	How many people refused to participate or dropped out? Reasons?	2 students signed up to a focus group but did not attend due to other commitments.
	Setting	
14. Setting of data collection	Where was the data collected? e.g. home, clinic, workplace	Data were collected online (video-conferencing platform)
15. Presence of non-participants	Was anyone else present besides the participants and researchers?	No.
16. Description of sample	What are the important characteristics of the sample? e.g. demographic data, date	Students: self-isolated/not, COVID-tested/not, living circumstances (on/off campus), year of study. Staff: role
	Data collection	
17. Interview guide	Were questions, prompts, guides provided by the authors? Was it pilot tested?	No
18. Repeat interviews	Were repeat interviews carried out? If yes, how many?	One staff interview was repeated due to issues with the interview recording
19. Audio/visual recording	Did the research use audio or visual recording to collect the data?	Focus groups and interviews were audio-recorded using a video-conferencing platform. Interviewers kept cameras on to assist with establishing rapport.
20. Field notes	Were field notes made during and/or after the interview or focus group?	Yes.

21. Duration	What was the duration of the interviews or focus group?	Approx. 60-90 mins.
22. Data saturation	Was data saturation discussed?	Yes.
23. Transcripts returned	Were transcripts returned to participants for comment and/or correction?	No – due to time sensitivity of the research
**Domain 3: analysis and findings**		
	Data analysis	
24. Number of data coders	How many data coders coded the data?	One, with a second researcher checking coding.
25. Description of the coding tree	Did authors provide a description of the coding tree?	No, however initial coding was informed by the interview guide, and coding was continuously refined.
26. Derivation of themes	Were themes identified in advance or derived from the data?	Themes were derived from the data.
27. Software	What software, if applicable, was used to manage the data?	NVIVO
28. Participant checking	Did participants provide feedback on the findings?	No
	Reporting	
29. Quotations presented	Were participant quotations presented to illustrate the themes / findings? Was each quotation identified? e.g. participant number	Yes
30. Data and findings consistent	Was there consistency between the data presented and the findings?	Yes
31. Clarity of major themes	Were major themes clearly presented in the findings?	Yes
32. Clarity of minor themes	Is there a description of diverse cases or discussion of minor themes?	Yes

Supplementary File 3: Student and staff interview guides

Student Focus Group Guide

About You (Interview Sample Characteristics)

Have you taken part in a university COVID-19 testing programme or had a COVID-19 test elsewhere?

Are you currently self-isolating? If so, why?

Have you isolated more than once?

Were you told to self-isolate after receiving a university asymptomatic test?

Did you go home over the winter break or were you staying in university accommodation during this time?

Impact of self-isolation

What are your views on self-isolation? Is it important, or not?

How did you feel when you learned you needed to isolate? How do you think other students' experiences of self-isolating have been?

How did being in self-isolation impact you?

Barriers and enablers to self-isolation

What are some things that made going into self-isolation easier? What helped you most during isolation?

Were there any challenges to going into isolation? Anything that made it difficult during your time in isolation?

Did you feel you were able to adhere to the isolation? If no, why not? What would have helped?

What would encourage students to self-isolate? What might discourage students from adhering to self-isolation advice?

Are there any particular groups that would find it harder than others, and why?

Support needs during isolation

What were some practical things that you needed or would have liked during isolation? Can you think of other things that would have been helpful?

Did you use any services or seek out support for your mental wellbeing during self-isolation?
What helped? Are there other supports you think would have been helpful?

What was the support like from your institution during self-isolation? Was there anything the university did that was really helpful or could have done differently?

How can the university best support students during periods of self-isolation? What are the key things that would be helpful for yourself or others?

Staff Interview Guide

What is your current job role?

In your role, do you have face-to-face contact with students?

Do you have other types of direct student contact? In what capacity?

Are you connected to the university COVID-19 testing services? Asymptomatic testing programme or on-campus health centre testing (Pillar 2)?

Have you had to self-isolate yourself? Do you have any other experience with COVID-19?

Response, adherence, impacts

How do you think students generally respond when they're told they have to self-isolate?

Do they think students are generally being adherent to self-isolation advice or not? (If yes or no, then why)

Does students' response to, adherence to, or the broader impact of self-isolating differ according to the reason students need to self-isolate (triggered by self or others)?

Does students' response to, adherence to, or the broader impact of self-isolating alter students' engagement with asymptomatic (or other) testing?

How do you think students are impacted during periods of self-isolation?

Has any of this changed, or might it change, during different periods of pandemic and with different government restrictions or university regulations?

Mental health and wellbeing

Have you noticed any particular mental health impacts in students who have to self-isolate? Through the pandemic more broadly? Has this raised any particular concerns?

Have you noticed any changes to students' mental health during different periods of pandemic? Might you expect changes in the future?

Barriers and facilitators:

What do you think can be done to make it easier for students to cope during self-isolation (and adhere to it) - both university-level initiatives in a higher education setting, but also more broadly for supporting young people)?

What factors might discourage students from adhering to self-isolation advice?

Are there any particular groups that might find it harder to adhere and why?

Support needs:

Do you know what is in place to support students who are self-isolating? What do students tend to access and why?

Which support approaches work and which don't? Why?

Are there any barriers for staff in supporting students who are self-isolating?

Are there any barriers for staff in supporting students with mental health concerns?

What else could be done?

Supplementary file 4. Thematic map illustrating the relationships between key themes and subthemes

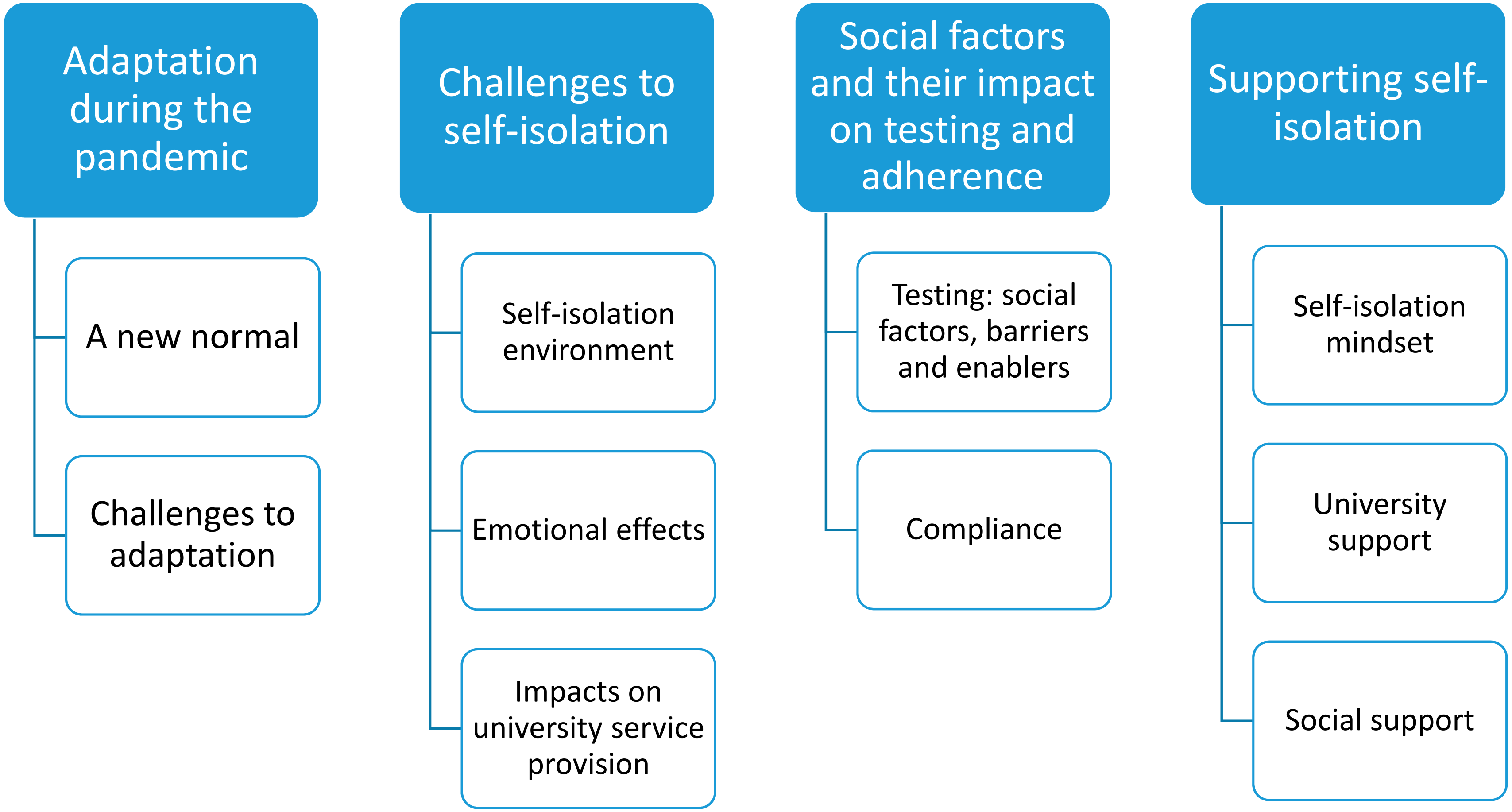

Supplement: Supplementary file 1 [file ijerph-18-10675-s001.zip › ijerph-1393212-supplementary.pdf]
